# ElectronixTutor: an intelligent tutoring system with multiple learning resources for electronics

**DOI:** 10.1186/s40594-018-0110-y

**Published:** 2018-04-16

**Authors:** Arthur C. Graesser, Xiangen Hu, Benjamin D. Nye, Kurt VanLehn, Rohit Kumar, Cristina Heffernan, Neil Heffernan, Beverly Woolf, Andrew M. Olney, Vasile Rus, Frank Andrasik, Philip Pavlik, Zhiqiang Cai, Jon Wetzel, Brent Morgan, Andrew J. Hampton, Anne M. Lippert, Lijia Wang, Qinyu Cheng, Joseph E. Vinson, Craig N. Kelly, Cadarrius McGlown, Charvi A. Majmudar, Bashir Morshed, Whitney Baer

**Affiliations:** 1 Institute for Intelligent Systems, University of Memphis, Memphis, USA; 2 Honorary Research Fellow, University of Oxford, Oxford, UK; 3 Institute for Creative Technologies, University of Southern California, Los Angeles, USA; 4 School of Computing, Informatics and Decision Systems Engineering, Arizona State University, Tempe, USA; 5 Department of Mathematical Sciences, Worcester Polytechnic Institute, Worcester, USA; 6 Department of Computer Science, Worcester Polytechnic Institute, Worcester, USA; 7 Department of Information and Computer Sciences, University of Massachusetts Amherst, Amherst, USA; 8 Department of Electrical and Computer Engineering, The University of Memphis, Memphis, USA

**Keywords:** ASSISTments, AutoTutor, Dragoon, Electronics, Intelligent tutoring systems, System integration

## Abstract

**Background:**

The Office of Naval Research (ONR) organized a STEM Challenge initiative to explore how intelligent tutoring systems (ITSs) can be developed in a reasonable amount of time to help students learn STEM topics. This competitive initiative sponsored four teams that separately developed systems that covered topics in mathematics, electronics, and dynamical systems. After the teams shared their progress at the conclusion of an 18-month period, the ONR decided to fund a joint applied project in the Navy that integrated those systems on the subject matter of electronic circuits. The University of Memphis took the lead in integrating these systems in an intelligent tutoring system called *ElectronixTutor*. This article describes the architecture of ElectronixTutor, the learning resources that feed into it, and the empirical findings that support the effectiveness of its constituent ITS learning resources.

**Results:**

A fully integrated ElectronixTutor was developed that included several intelligent learning resources (AutoTutor, Dragoon, LearnForm, ASSISTments, BEETLE-II) as well as texts and videos. The architecture includes a student model that has (a) a common set of knowledge components on electronic circuits to which individual learning resources contribute and (b) a record of student performance on the knowledge components as well as a set of cognitive and non-cognitive attributes. There is a recommender system that uses the student model to guide the student on a small set of sensible next steps in their training. The individual components of ElectronixTutor have shown learning gains in previous decades of research.

**Conclusions:**

The ElectronixTutor system successfully combines multiple empirically based components into one system to teach a STEM topic (electronics) to students. A prototype of this intelligent tutoring system has been developed and is currently being tested. ElectronixTutor is unique in its assembling a group of well-tested intelligent tutoring systems into a single integrated learning environment.

## Background

Intelligent tutoring systems have been developed for nearly four decades on many STEM topics after the field was christened with the edited volume, *Intelligent Tutoring Systems*, by Sleeman and Brown (1982). Intelligent tutoring systems (ITSs) are computer learning environments designed to help students master difficult knowledge and skills by implementing powerful intelligent algorithms that adapt to the learner at a fine-grained level and that instantiate complex principles of learning (Graesser et al. in press). An ITS normally works with one student at a time because learners have different levels of mastery, specific deficits in knowledge, and idiosyncratic profiles of cognitive and non-cognitive attributes.

ITS environments incorporate learning mechanisms that are a generation beyond conventional computer-based training. Conventional training systems sometimes adapt to individual learners, but they do so at a coarse-grained level with limited options (e.g., two to five) at each point in the student-system interaction. For example, the student (a) studies material presented in a lesson, (b) is tested with a multiple-choice test or another objective test with a small number of options, (c) receives feedback on the test performance, (d) re-studies the material in “a” if the performance in “c” is below threshold, and (e) progresses to a new topic if the performance in “c” exceeds the specified threshold. An ITS tracks the knowledge, skills, and other psychological characteristics of students at a finer grain size and adaptively responds to the student by applying computational mechanisms in artificial intelligence and cognitive science (Sottilare et al. 2014; VanLehn 2006; Woolf 2009). For an ITS, adaptivity is so fine-grained that most tutorial interactions on a topic follow a unique sequence.

ITSs have been developed for a wide range of STEM subject matters. Many have targeted mathematics and other well-formed, quantitatively precise topics. In the areas of algebra and geometry, for example, there are the *Cognitive Tutors* (Aleven et al. 2009; Koedinger et al. 1997; Ritter et al. 2007) and *ALEKS* (Falmagne et al. 2013); one assessment compared these two systems on learning gains and resulted in a virtual tie (Sabo et al. 2013). In the area of technology and engineering, there are ITSs on electronics (*SHERLOCK*, Lesgold et al. 1992; *BEETLE-II*, Dzikovska et al. 2014), digital information technology (*Digital Tutor*, Fletcher and Morrison 2012), and database retrieval (*KERMIT*, Mitrovic et al. 2007). In the area of physics, VanLehn and his colleagues have developed *Andes* (VanLehn 2011).

Some ITSs focus on knowledge domains that have a stronger verbal foundation as opposed to mathematics and precise analytical reasoning (Johnson and Lester 2016). *AutoTutor* and its descendants (Graesser 2016; Nye et al. 2014a, 2014b) help college students learn by holding a conversation in natural language. Conversational agents (also known as interactive agents and pedagogical agents) are a general class of learning environments that are either scripted or intelligently adaptive (Atkinson 2002; Craig et al. 2002; Johnson et al. 2000; Graesser and McNamara 2010; Moreno et al. 2001). Conversational agents have talking heads that speak, point, gesture, and exhibit facial expressions. They can guide the interaction with the learner, instruct the learner what to do, and interact with other agents to model ideal behavior, strategies, reflections, and social interactions (Craig et al. 2015; Graesser et al. 2014; Johnson and Lester 2016; Kim et al. 2007). These agents have been designed to represent different human instructional roles, such as experts (Johnson et al. 2000; Kim and Baylor 2016), tutors (Nye et al. 2014a, 2014b), mentors (Baylor and Kim 2005; Kim and Baylor 2016), and learning companions (Chan and Baskin 1990; Dillenbourg and Self 1992; Goodman et al. 1998). Research supports the idea that conversational agents have a positive effect on learning (Schroeder et al. 2013; Schroeder and Gotch 2015).

Some conversational agents are not merely scripted but attempt to understand the natural language of the learner and adaptively respond with intelligent mechanisms. Examples of these intelligent conversation-based systems have covered STEM topics such as computer literacy (Graesser et al. 2004), physics (*DeepTutor*, Rus et al. 2013; *AutoTutor*, VanLehn et al. 2007), biology (*GuruTutor*, Olney et al. 2012), and scientific reasoning (*Operation ARIES/ARA*, Halpern et al. 2012; Kopp et al. 2012; Millis et al. in press). Other examples of systems with intelligent conversational agents that have successfully improved student learning are *MetaTutor* (Lintean et al. 2012), *Betty’s Brain* (Biswas et al. 2010), *Coach Mike* (Lane et al. 2011), *iDRIVE* (Craig et al. 2012; Gholson et al. 2009), *iSTART* (Jackson and McNamara 2013; McNamara et al. 2006), *Crystal Island* (Rowe et al. 2011), *My Science Tutor* (Ward et al. 2013), and *Tactical Language and Culture System* (Johnson and Valente 2009).

Reviews and meta-analyses confirm that ITS technologies frequently improve learning over classroom teaching, reading texts, and/or other traditional learning methods. These meta-analyses typically report effect sizes (signified by *d*), which refer to the difference between the ITS condition and a control condition, calibrated in standard deviation units. The reported meta-analyses show positive effect sizes that vary from *d* = 0.05 (Dynarsky et al. 2007; Steenbergen-Hu and Cooper 2014) to *d* = 1.08 (Dodds and Fletcher 2004), but most hover between *d* = 0.40 and *d* = 0.80 (Kulik and Fletcher 2015; Ma et al. 2014; Steenbergen-Hu and Cooper 2013; VanLehn 2011). A reasonable meta-meta estimate from all of these meta-analyses is *d* = 0.60. This performance is comparable to human tutoring, which varies between *d* = 0.42 (Cohen et al. 1982) and *d* = .80 (VanLehn 2011), depending on the expertise of the tutor. Human tutors have not varied greatly from ITSs in direct comparisons between ITSs and trained human tutors (Graesser 2016; Olney et al. 2012; VanLehn 2011; VanLehn et al. 2007).

The subject matter being tutored limits the magnitude of the learning gains from ITS. For example, it is difficult to obtain high effect sizes for literacy and numeracy because these skills are ubiquitous in everyday life and habits are automatized. In contrast, when the student starts essentially from square one, effect sizes should be more robust. As a notable example, the *Digital Tutor* (Fletcher and Morrison 2012; Kulik and Fletcher 2015) improved information technology knowledge by an effect size as high as *d* = 3.70 and *d* = 1.10 for skills. Such large effect sizes would be unrealistic for basic literacy and numeracy.

The US Department of Defense has historically played a major role in funding efforts to develop ITS technologies (Chipman 2015). The DoD recognized the need to develop training systems capable of promoting deeper learning on STEM areas that could not be delivered reliably within conventional learning environments. The Office of Naval Research (ONR) consistently supported these research efforts for many decades. More recently, the Army Research Laboratories spearheaded the Generalized Intelligent Framework for Tutoring (Sottilare et al. 2013; www.gifttutoring.org) to scale up these systems for more widespread use. The Advanced Distributed Learning community (2016) has promoted standards for developing and integrating systems. The National Science Foundation (NSF) and Institute for Education Sciences have supported ITSs since the turn of the millennium, as exemplified by the NSF-funded Pittsburgh Science of Learning Center (Koedinger et al. 2012).

One of the persistent challenges with ITS is that it takes a large amount of time and funding to develop these systems and to tune their complex adaptive models through iterative empirical testing. The field has attempted, over many decades, to reduce the development time and cost through authoring tools (Murray et al. 2003; Sottilare et al. 2015). The ideal vision is that an expert on a STEM topic, but without advanced computer expertise, would be able to use authoring tools to provide content on any particular STEM topic and for the tools to generate a complete and runnable ITS from the authored content alone. Although progress in those efforts has resulted in modest reductions in time and costs, the complex intersection of content, pedagogical expertise, and programming expertise that is needed to create an ITS has continued to hinder major reductions in the speed and costs of development.

With this context in mind, ONR launched the STEM Challenge initiative for teams to develop and test an ITS on any STEM topic in a limited amount of time (18 months). From several dozen applications, four teams were selected: The University of Memphis, Arizona State University, BBN/Raytheon, and a collaboration between the University of Massachusetts and Worcester Polytechnic Institute. These teams reported their findings and competed for another round of funding to focus on a Navy-relevant STEM area. The ONR selected analog electronic circuits as the subject matter for the second wave of funding. The University of Memphis was selected to take the lead in developing an ITS system on electronic circuits but with a view to design the system so that it would integrate electronics content developed by the other teams. In essence, the ITS would be an ensemble of ITSs developed by multiple teams on the same topic. Consequently, with The University of Memphis serving as the lead, *ElectronixTutor* was developed within 18 months, integrating intelligent learning resources provided by all four teams—a unique undertaking in the history of the ITS field.

We are currently collecting empirical data on ElectronixTutor, so empirical findings on learning gains and usage patterns are not yet available. However, each of the core components of ElectronixTutor has been empirically validated across a number of studies, giving us confidence in the efficacy of the system which encapsulates them. There are two primary goals of this article. First, we describe ElectronixTutor and the individual ITS learning resources that form the system (i.e., those developed by the four teams). Second, we review empirical evidence for learning gains on STEM topics that were developed by these teams and applied to the development of ElectronixTutor.

## Results

### Overview of ElectronixTutor

ElectronixTutor focuses on Apprentice Technician Training courses in electronics for Navy trainees who have completed boot camp and are in the process of A-school training conducted by the Navy Educational Training Command. These individuals have above-average scores in the Armed Services Vocational Aptitude Battery, so they have the cognitive capacity to learn electronics. They currently take courses led by a human instructor in a traditional classroom that includes lectures, reading materials, hands-on exercises with circuit boards, and occasional access to human tutors. An instructor typically teaches 25 sailors at a time for 8 h a day for 8 to 12 weeks. ElectronixTutor aims to supplement the classroom instruction with advanced learning environments (ITS and other forms of adaptive learning technologies) that can help the sailors achieve the instructional objectives more efficiently.

### Learning resources

ElectronixTutor integrates many learning resources into one system. Some of the learning resources are based on research in ITSs, whereas others are conventional resources that are not adaptive to the learner’s idiosyncratic profile of knowledge, skills, and abilities but can be orchestrated by ElectronixTutor for a more adaptive experience. We first discuss the ITS-based learning resources, which are reviewed here in brief; these are discussed in more detail in subsequent sections, including empirical evidence for their influence on learning gains.

*AutoTutor* has the option for one or two conversational agents (i.e., computer-generated talking heads) to promote verbal reasoning, question answering, conceptual understanding, and natural language interaction (Graesser 2016; Graesser et al. 2014; Nye et al. 2014a, 2014b). Deep questions (e.g., “why?,” “how?,” and “what if”; Graesser and Person 1994) are asked by a tutor agent, followed by a multi-turn conversation that is adaptive based on the quality of the student’s responses. The main questions range from broad questions that require several ideas in the answer to more focused questions that address a specific idea. The University of Memphis took the lead on developing AutoTutor.

*Dragoon* has a mental model construction and simulation environment (VanLehn et al. 2016a, 2016b; Wetzel et al. 2016). The Dragoon system provides instructional support to help the student construct mental models of circuits with nodes and relations. The student can click on circuit elements and see how changing their values affects the system as a whole. Arizona State University took the lead in developing the Dragoon ITS.

*LearnForm* is a general learning platform that is used for the creation and delivery of learning tasks that require problem-solving. A problem (learning task) consists of a student being presented with a problem statement, multiple-choice questions, feedback, and finally a summary of a correct answer. The student is free to select the problems to work on, so the system allows self-regulated learning. However, in ElectronixTutor, the problems are systematically assigned under specific conditions that reflect intelligent task selection. Raytheon/BBN took the lead on developing the LearnForm problems.

*ASSISTments* is a platform for developing subject matter content, assessment materials, and other learning technologies on the web (Heffernan and Heffernan 2014). ASSISTments played an early role in integrating the learning resources because it had an organized learning management system for incorporating viewpoints from teachers, students, and resource developers. The major ITS component is “skill building” on the mathematics of Ohm’s and Kirchhoff’s laws, which are fundamental to electronics reasoning. Worcester Polytechnic Institute took the lead on ASSISTments and skill building.

*BEETLE-II* is a conversation-based ITS that was previously funded by the ONR on basic electricity and electronics (Dzikovska et al. 2014). BEETLE-II was pitched at a basic, lower-level understanding of circuits, such as open and closed circuits, voltage, and using voltage to find a circuit fault. BEETLE-II improved learning, but it was at the macro-level of discourse and pedagogy rather than the micro-level language adaptation. Therefore, the curriculum and macro-discourse level was incorporated into ElectronixTutor. The Naval Air Warfare Center Training Systems Division provided this content.

A number of conventional learning resources were included in ElectronixTutor in addition to these intelligent, adaptive, and well-crafted ITS learning resources. The conventional resources are not adaptive, but they are under the complete control of the student when studying the material. Thus, they can be especially helpful for students who prefer the free selection and study of materials (i.e., self-regulated learning).

#### Reading documents

ElectronixTutor includes ample traditional, static documents, including 5000 pages of the Navy Electronics and Electricity Training Series (U.S. Navy 1998), the Apprentice Technical Training (ATT) PowerPoints used by the instructors, and an overview of major electronics concepts that was prepared by the ASU team.

#### Viewing videos

ElectronixTutor automatically presents specific video lessons under various conditions or alternatively permits the student to voluntarily access the material. Some of these videos instruct the students on subject matter content but others train the students on using the learning resources.

#### Asking questions and receiving answers through Point & Query

In the AutoTutor system, each main question is accompanied by a figure, and each figure may contain one or more “hot spots.” When the trainee clicks on a hot spot, a menu of questions appears, the trainee selects a question from the menu, and the answer is presented. Although students tend to ask few questions in the classroom and tutoring environments (Graesser and Person 1994; Graesser et al. 2005), the nature of the questions being asked is diagnostic of student understanding (Graesser and Olde 2003). Point & Query has been shown to increase the frequency and diversity of questions (Graesser et al. 2005).

The above learning resources are designed to accommodate particular learner profiles. The broad AutoTutor questions and mental model constructions of Dragoon are ideal for students at higher levels of mastery. The skill builders, BEETLE, Point & Query, readings, and videos target students at lower levels of mastery. The focused AutoTutor questions and LearnForm multiple-choice questions are ideal for intermediate states of mastery. These particular learning resources are orchestrated and recommended by ElectronixTutor on the basis of the student’s performance on tasks during learning—not through any pretest. We further examine these recommendations later when we discuss the Recommender System.

### Topics and knowledge components

The ElectronixTutor team followed the *Topic* + *Knowledge Component* framework proposed by researchers at the Pittsburgh Science of Learning Center (Koedinger et al. 2012). This framework is a principled approach to guiding recommendations on topics to be covered. Sometimes, the course curriculum guides the recommended topics, as reflected in a syllabus or day-by-day outline of content covered. At the other end of the continuum, self-regulated learning is available, wherein the students select the topics they want to cover in whatever order they wish. In between, topics can be recommended by an intelligent *Recommender System* that considers the history of a student’s performance and psychological attributes. For all of these approaches, an organized set of subject matter topics and knowledge components needs to be considered in the representation of the domain knowledge.

VanLehn at ASU prepared a document that covers the following scope and sequence of topics based on the Navy curriculum at A-school: circuit analysis, Ohm’s law, series circuits, parallel circuits, PN junctions, diode limiters, diode clampers, transistors, CE amplifiers, CC amplifiers, CB amplifiers, multistage amplifiers, and push-pull amplifiers. That being said, the Navy and individual instructors may have different visions on the selection of topics and the order of topics in the course. Such changes can be made in the *Course Calendar* facility of ElectronixTutor.

A more fine-grained specification of electronic circuit knowledge in ElectronixTutor consists of *knowledge components*. A topic has an associated set of these knowledge components (KCs). Each topic included at least three KCs to cover the *structure* of the circuit (or its physics, if the component is a primitive), its *behavior*, and its *function* (i.e., what it is typically used for). Example knowledge components for a transistor are CE transistor behavior, CC transistor function, and CE push-pull amplifier structure. The system is not strictly hierarchical because one KC can be linked to multiple topics. Mastery of each KC is assessed by the various learning resources. A particular learning resource may or may not address a particular KC so there is only partial overlap among learning resources in covering the KCs. To the extent that learning resources overlap, we are able to reconstruct, through data mining procedures, which learning resource (LR) is best tailored to particular KCs for particular categories of learners (L). This is essential for determining the right content to present to the right learner at the right time, which is one of the mantras of learning technologies. Consequently, the KC × LR × L matrix was part of the task analysis of ElectronixTutor.

As students work on problems, their performance on topics and KCs is tracked and retained in a *Learning Record Store*. In essence, the *Student Model* (i.e., the cognitive and other psychological attributes considered by the ITS) consists of the data stored in the Learning Record Store. Some of the content addresses subject matter knowledge (i.e., topics and KCs), but other content addresses generic characteristics that range from verbal fluency to grit (i.e., persistence on difficult tasks). Each of the intelligent learning resources therefore needs to assess the student’s performance on the relevant KCs associated with the topics. Students attempt tasks and the performance scores on each KC are recorded.

### ElectronixTutor software architecture

The integration of the learning resources and Recommender System experienced some changes throughout the project when we leveraged an ongoing companion project between USC, ASU, and Memphis called the *Personal Assistant for Life-Long Learning: PAL3* (Swartout et al. 2016). The design process for the ElectronixTutor architecture required generalizable solutions for integrating multiple pre-existing ITSs and conventional learning resources into a coherent user experience. In particular, this effort integrated learning resources from AutoTutor, Dragoon, LearnForm, BEETLE, ASSISTments, Point & Query, readings, and videos. Performance needed to be updated in the Student Model and associated Learning Record Store. Recommendations on the next learning resource and topic to cover needed to be addressed, based on local and global recommendations by ElectronixTutor. Integrating multiple asynchronous intelligent systems presented serious challenges: (1) the system control is distributed rather than governed by a single ITS, (2) different learning resources provide and require different kinds of information, and (3) data from different learning resources need to influence actionable representations for a Student Model and Recommender System.

#### Distributed system control: using SuperGLU for real-time coordination

We leveraged the SuperGLU (Generalized Learning Utilities) open-source framework as one approach to handling the problem of integrating and coordinating distributed web-based systems in real time (Nye 2016). This framework was designed with the purpose of integrating intelligent systems for real-time coordination, with the first application integrating a commercial mathematics adaptive learning system with AutoTutor (Nye et al. 2014a, 2014b). It is beyond the scope of this article to cover the technical implementation of SuperGLU in detail. However, one major piece of functionality consisted of building a recommender service that could call particular learning resources by analyzing features from the KC × LR × L matrix and the student’s profile in the Learning Record Store. Moreover, the framework accommodated a single page in a web browser that communicated simultaneously with an average of four different web servers when ElectronixTutor was running. This configuration works as long as the communication is stable among constituent parts.

**Fig. 1:**
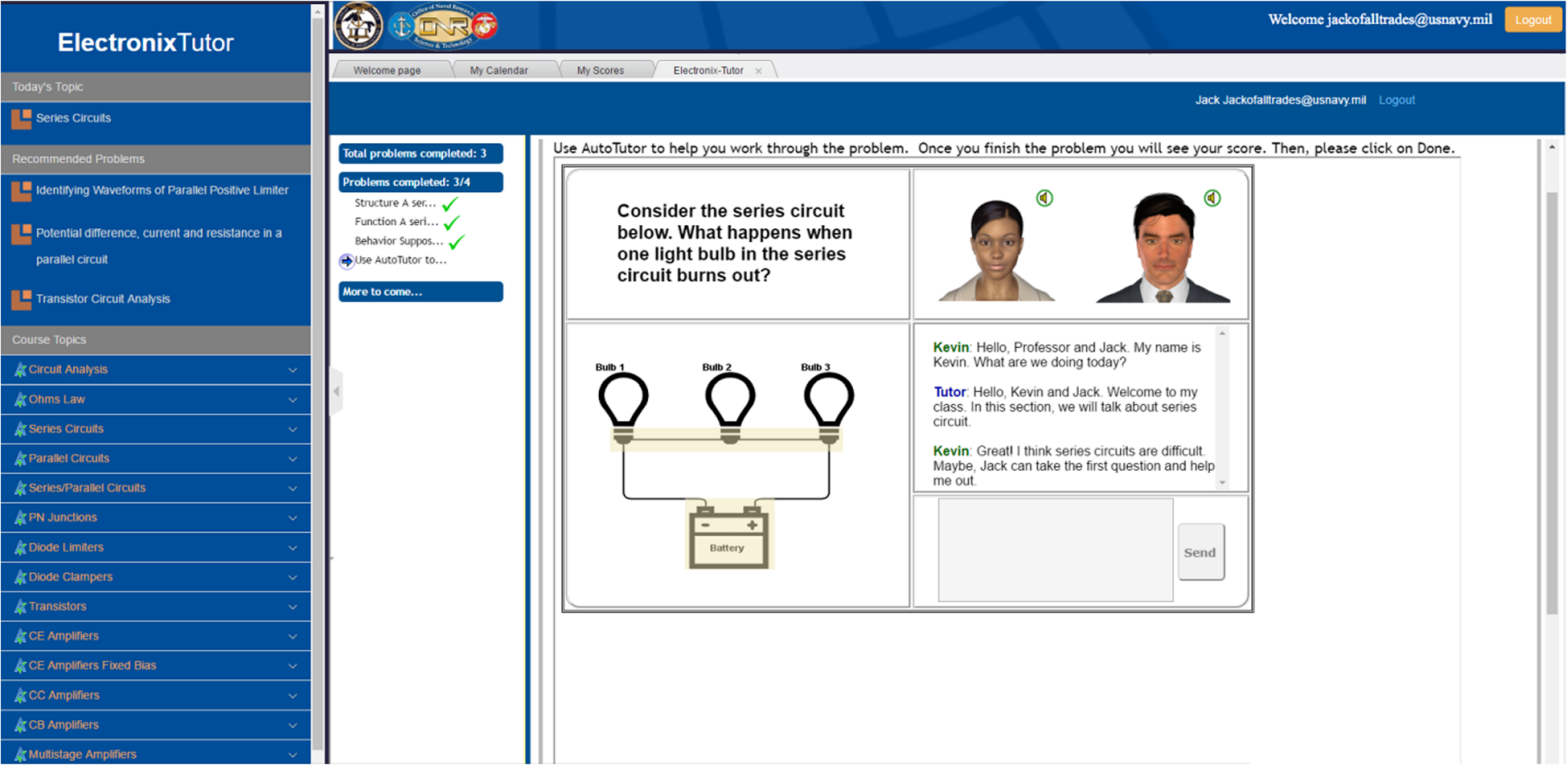
ElectronixTutor user interface snapshot

Figure 1 shows a screenshot of ElectronixTutor during an interaction with AutoTutor. It is the layout of information on the screen that is important for the present purposes rather than details of the content. The left part of the window shows a list of topics to be covered. The topic at the top is *Today’s Topic* that is generated by the instructor’s Course Schedule. Three *Recommended Problems* are next presented based on tracking the long-term performance of the individual student and also upcoming topics in the curriculum. Finally, the total set of *Course Topics* is listed that self-regulated learners can pursue.

The information in the main center-right area of the display depends on the tab which is active on the ribbon above it. Four different types of information can be displayed, depending on which tab is selected. The order of tabs, from left to right is The initial welcome screen (“Welcome Page”), a calendar of topics and resources (“My Calendar”), performance scores that the learner can view (“My Scores”), and a problem being worked on (in this cause an AutoTutor problem). The *My Calendar* tab displays a curriculum schedule for the topics and resources; it serves as an alternate view for seeing how the content on the left panel aligns with a course or personal curriculum. In Fig. 1, the two AutoTutor conversational agents appear in the context of a problem to be solved. These two agents hold a conversation with the student during the course of solving the problem, as will be described in the "AutoTutor" section. When an activity is completed, it reports a summary score and, optionally, more fine-grained performance data such as hints, feedback, and other events.

In the *My Scores* tab, the student receives feedback on how he or she is doing. A list of knowledge components (KCs) and horizontal bars are presented that indicate the student’s progress on each KC (ranging from 0 to 1). These scores reflect performance on the learning resources that contributed to the particular KC. To accomplish this computation, each learning resource needs to contribute a score (0 to 1) whenever a resource is recruited and involves the KC. The current version of ElectronixTutor assigns an equal weight to each KC and also each learning resource (i.e., AutoTutor, Dragoon, LearnForm, ASSISTments) that assesses the student’s performance on a particular KC. However, as data are collected from students, the weights will be adjusted to fit outcome measures that assess learning of the particular KCs. Eventually, the overall KC score will be weighted average of opportunities for measurement and the learning resources that contributed to the measurement; alternatively, only the recent performance on a KC will be scored to get an updated performance measure. Since our learning resources are of very different types (conversation, simulation, multiple choice, etc.), we cannot assume that all resources contribute to the assessment equally. The Recommender System examines these performance scores and opportunity history to suggest topics that have lower mastery or have not been visited recently.

Each of the main topics has a topic “bundle,” which is a conditional branching structure composed of various learning resources that cover that specific topic (along with the associated KCs). In the initial prototype, ElectronixTutor used ASSISTments to govern selection of learning resources within a topic bundle. That is, ASSISTments provided “If-Then” functionality within a bundle to sequence learning resources and tasks within a topic, based on performance of the student on the specific topic and also previous measures in the Learning Record Store. The conditional branching between different learning resources within a topic is sensitive to the scores while tracking student knowledge at the KC level and governs what learning resources are presented at the local bundle level (as opposed to a more global level).

Below is an example local bundle template that illustrates conditional branching for a particular topic. Once again, this local branching structure for one topic is different from the global recommendations that are delivered by the Recommender System (which is not governed by a single topic).

(0) Read a succinct summary of a topic for as long as the student wishes.
(1) Present a broad AutoTutor question that targets multiple knowledge components (KCs) associated with a topic.
(2) If the performance in #1 meets or exceeds a high threshold, then assign a Dragoon problem.
(3) If the performance in #1 is below the high threshold, then assign an AutoTutor knowledge check question that targets a single KC.
(4) If the performance of #1 and #3 is above the medium threshold, then assign the LearnForm problems.
(5) If the performance of #1 and #3 is below the medium threshold, then assign either the readings, BEETLE, or the skill builder depending on the psychological characteristics in the Learning Record Store, such as verbal fluency, electronics knowledge, and/or numeracy.

The above decision rules serve as an example of how decisions are made within a bundle, but there are other alternative models of local decision-making that will be explored in the future.

An example illustrates the experience of a student learning with ElectronixTutor. When the student is assigned a topic (such as rectifiers), the student is assigned a rectifier bundle of learning resources that begins with a succinct summary description to be read. After this initial reading about the topic, an AutoTutor “Deep Reasoning” question is presented that assesses the student on relevant KCs associated with the summary description and topic bundle. This student may have a strong understanding of several KCs within the topic but lack comprehension on one of them. AutoTutor would recognize this deficiency and respond accordingly, suggesting that the student engage in an AutoTutor “Knowledge Component” problem that specifically targets that missing KC. For example, one AutoTutor Knowledge Component problem could ask the student “What is the main function of a rectifier circuit?” and the student is expected to provide the answer “It converts an AC signal into a DC signal.” The AutoTutor conversation includes hints and other questions to encourage the student to express particular ideas, phrases, and words, as will be discussed later.

Suppose the student fails to demonstrate proficiency in answering the AutoTutor questions, the conditional branching would suggest a different, low-level type of learning resource, such as BEETLE-II or the NEETS readings. This would hopefully help the student learn the basic information. The student would subsequently receive additional AutoTutor questions and branch to the intermediate-level LearnForm questions, or ultimately to the very challenging Dragoon problems. Altogether, this single topic bundle could take an hour or even longer to the extent that the student struggles with multiple KCs associated with the topic.

#### Recommender System

The Recommender System is a separate mechanism for generating recommendations to the student on what to do next. The Recommender System recommends topics and learning resources based on the student’s past long-term performance and psychological profile. The Recommender provides three main functions: student model estimates of learning, personalized recommendations for learning tasks to complete, and the ability to store, retrieve, and modify a calendar that schedules both topics and individual learning tasks. In total, these capabilities give three ways for a learner to consider a learning task: the overview panel that lists all available tasks, the currently recommended resources, and the calendar of resources. This redundancy was intentionally designed to serve different pedagogical use cases. In a typical strictly paced classroom environment, the overview and calendar are expected to be central elements; the recommendations are expected to be used either during designated class time or to support students who are significantly ahead or behind the rest of the class. On the converse, for self-regulated study, the recommendations serve a more central role in helping learners move through the material efficiently but under their own control. In between these two extremes is the intelligent set of recommendations, wherein the student can choose and thereby allow some semblance of agency.

**Fig. 2:**
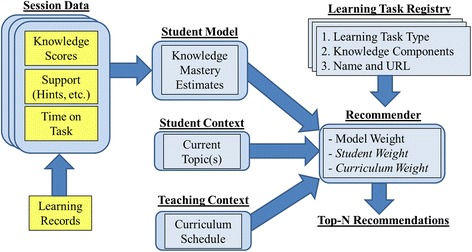
Integrating student model estimates and learning task recommendations

The high-level information flow for these capabilities is shown in Fig. 2. Due to user data not yet being available, each of these functions was implemented based on heuristic metrics, but it will be straightforward to substitute these models with more complex models in the future. The student model currently considers three types of features: performance scores reported by tasks, scaffolding support reported by tasks, and time required to complete a task. All of these features are derived from task sessions that are constructed dynamically from the learning records logged from the messages described in the Appendix. Performance scores consider two types of messages: *Completed* and *KnowledgeComponentScore* (i.e., a KC score). The *Completed* message reports an overall performance on a task or topic, which the student model by default assumes is the performance on all KCs known to apply. The ITS may modify raw KC scores for a task by the level of support provided to the learner (more hints and negative feedback reduce the score) and the amount of time spent to achieve that score (time after a certain threshold incurs a small penalty). If the ITS sends a *KnowledgeComponentScore* during the same session for that task, it will update any previous scores for that task and relevant resource. Across different sessions of tasks, student model estimates for each KC are currently calculated using a simple exponential moving average of scores that weights recent experiences higher than earlier experiences.

The Recommender System ranks learning tasks that the student should attempt based on the Student Model and a novelty calculation. From the Recommender’s standpoint, the student model provides scores for each KC between 0 and 1, representing mastery of the student’s knowledge of that skill or information. At present, because the model is anticipated to help remediate weaknesses in learner knowledge, resources are scored based on their potential average learning gain (e.g., the amount their KCs could improve if they performed perfectly on that resource). Novelty is determined by an exponential decay function of the number of attempts on each resource, so that the Recommender prefers suggesting new resources more than re-attempting others. Finally, some functionality was designed for the Recommender to consider the curriculum calendar and current topics the student was recently studying. However, this functionality is shown in italics because it is not used in the current prototype that generates recommendations.

## Intelligent tutoring system learning resources

This section describes each of the learning resources in greater detail, including available evidence that the ITS mechanisms promote learning gains. The empirical findings refer to previously developed systems that have been tested on various STEM subject matters (as opposed to the content of ElectronixTutor). However, learning gains are expected in ElectronixTutor, given the successes of these ITSs on previous STEM subject matters.

### AutoTutor

AutoTutor helps students learn through conversational agents that communicate with the students in natural language and thereby co-constructs answers to questions or solutions to problems. These constructive and interactive activities in natural language encourage deeper comprehension according to particular principles of learning. Empirical evidence supports the claim that AutoTutor and similar computer tutors with natural language dialog yield learning gains comparable to trained human tutors on STEM subject matters, with effect sizes averaging *d* = 0.8, ranging from of 0.3 to 2.0 (Graesser 2016; Kopp et al. 2012; Nye et al. 2014a, 2014b; Olney et al. 2012; Rus et al. 2013; VanLehn 2011; VanLehn et al. 2007).

Sometimes it is better to have two conversational agents, namely a tutor agent and a peer agent, in what is called a *trialogue* (Graesser et al. 2014). The student can observe the tutor agent and peer agent interact to model good behavior, which is sometimes helpful for students with low knowledge and skills. The more advanced student can attempt to teach the peer agent, with the tutor agent stepping in as needed. The two agents can disagree with each other and thereby stimulate cognitive disequilibrium, productive confusion, and deeper learning (D’Mello et al. 2014). Trialogues were implemented in the original STEM Challenge grant in the area of algebra with an ITS called ALEKS (Falmagne et al. 2013). Trialogues are also used in ElectronixTutor.

AutoTutor presents problems to solve and difficult questions to answer that require reasoning and that cover one to seven sentence-like conceptual expressions (e.g., semantic propositions, claims, main clauses) in an ideal response. The human and agents co-construct a solution or answer by multiple conversational turns. It may take a dozen to a hundred conversational turns back and forth to solve a problem or answer a difficult question. AutoTutor also has a talking head that speaks, gestures, and exhibits facial expressions.

It is beyond the scope of this article to describe the mechanisms of AutoTutor in detail (see Graesser 2016; Nye et al. 2014a, 2014b). However, an important feature is a systematic conversational mechanism called *expectation and misconception-tailored (EMT) dialog* (or trialogue). A list of *expectations* (anticipated good answers, steps in a procedure) and a list of anticipated *misconceptions* (bad answers, incorrect beliefs, errors, bugs) are associated with each task. As the students articulate their answers over multiple conversational turns, the contents of their contributions are compared with the expectations and misconceptions. Students rarely articulate a complete answer in the first conversational turn, but rather, their answers are spread out over many turns as the tutor generates hints and other conversational moves to enable the students to express what they know. The students’ answers within each turn are typically short (one to two speech acts on average), vague, ungrammatical, and not semantically well-formed. AutoTutor compares the students’ content to expectations and misconceptions through pattern-matching processes with semantic evaluation mechanisms motivated by research in computational linguistics (Rus et al. 2012).

Rather than simply lecturing to the student, the tutor provides scaffolding for the student to articulate the expectations through a number of dialog moves. A *pump* is a generic expression to get the student to provide more information, such as “What else?” or “Tell me more.” *Hints* and *prompts* are selected by the tutor to get the student to articulate missing content words, phrases, and propositions. A hint tries to get the student to express a lengthy constituent (e.g., proposition, clause, sentence), whereas a prompt is a question that tries to get the student to express a single word or phrase. The tutor generates an *assertion* if the student fails to express the expectation after multiple hints and prompts. AutoTutor provides a cycle of *pump* → *hint* → *prompt* → *assertion* for each expectation until the expectation is covered. As the student and tutor express information over many turns, the list of expectations is eventually covered and the main task is completed.

The student sometimes articulates misconceptions during the multi-turn tutorial dialog. When the student content has a high match to a misconception, AutoTutor acknowledges the error and provides correct information.

**Fig. 3:**
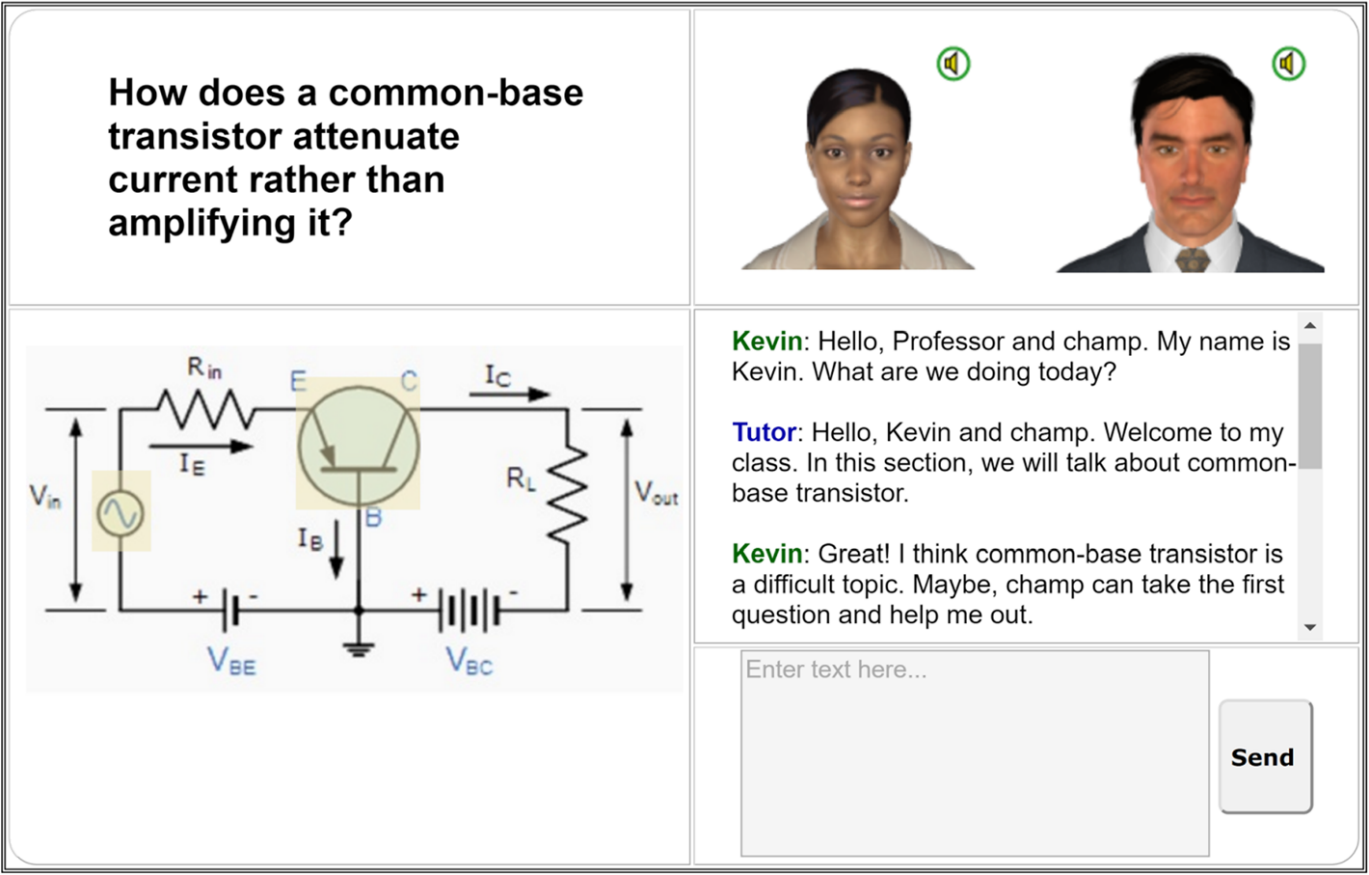
AutoTutor screenshot in ElectronixTutor

Figure 3 shows a screenshot of the main parts of the AutoTutor ITS within ElectronixTutor. The tutor and peer agent appear in the upper right. The main question asked by the tutor agent is printed in the upper left in addition to the tutor agent asking the question: “How does a common-base transistor attenuate current rather than amplify it?” A picture of a circuit is displayed in the lower left and the chat interaction is shown in the lower right of the screenshot. The chat facility shows the trialogue history plus an area where the human types in text (“Enter text here.”).

The trialogue attempts to get the student to express a number of expectations when the main question is asked. Below are some of the expectations and misconceptions for this question “How does a common-base transistor attenuate current rather than amplify it?”

- E1: The emitter current is greater than any other current in a common-base transistor.
- E2: The current gain of a common-base transistor is less than one.
- M1: The emitter current is lower than any other current in a common-base transistor.
- M2: The current gain of a common-base amplifier is greater than one.

As the trialogue conversation proceeds, AutoTutor matches the student contributions to these expectations and misconceptions. This is possible because of advances in computational linguistics (Jurafsky and Martin 2008; McCarthy and Boonthum-Denecke 2012) and statistical representations of world knowledge and discourse meaning, such as latent semantic analysis (Landauer et al. 2007). Indeed, the accuracy of these matches in AutoTutor is almost as reliable as trained human annotators (Cai et al. 2011). In order to handle misspellings and the scruffiness of natural language, the content of the expectations are represented as regular expressions as well as vector representations. For example, the following regular expression attempts to capture the phrase “less than one” in E2:

- \b(less|bott?[ou]m|decrea\w*|drop\w*|almos\w*|dip\w*|end\w*|low\w*|near\w*|small\w*|few\w*)\b, \b(on\w*?|1|unity)\b

Synonyms are provided for words and the first few letters of a word are sufficient for determining a match.

As discussed, AutoTutor attempts to get the student to articulate the expectations. Students are notoriously incomplete in articulating answers so AutoTutor agents provide pumps, hints, and prompts to encourage the student to articulate the content. For example, some of the hints and prompts for expectation E2 would be “What is the current gain of the transistor?”, “The current gain of the common-base transistor is less than what?”, and “What is less than one in the common-base transistor?” The selection of the hints and prompts attempts to elicit missing words and phrases in the expectation and thereby achieve pattern completion.

The primary pedagogical goal of AutoTutor is to encourage the student to verbally articulate content and steps in reasoning during the course of answering challenging questions or solving challenging problems. The desired content is captured in expectations. These expectations in turn are mapped onto the knowledge components (KCs) discussed earlier (Koedinger et al. 2012). There is also a non-hierarchical mapping (many-to-many) between the KCs and the main topics. The KCs and topics unite the curriculum and all of the learning resources in ElectronixTutor. Although AutoTutor measures mastery of KCs and topics through natural language in ElectronixTutor, AutoTutor also has the capacity to accommodate student actions that involve clicking, dragging, dropping, toggling, and even interactions in virtual worlds (Cai et al. 2015; Zapata-Rivera et al. 2015). In fact, simple clicks were emphasized in a project that helps struggling adult readers who have minimal abilities to type in written responses (Graesser et al. 2016).

### Dragoon

Dragoon is based on the hypothesis that a good way to understand a system is to construct a model of it, a hypothesis with considerable empirical support (VanLehn 2013). Moreover, constructing a model is not only a means to an end, namely understanding a system thoroughly, but also an end in itself, namely a cognitive skill that STEM learners should acquire. Modeling is one of only eight focal practices endorsed by the Next Generation Science Standards (NGSS 2013). Although “model” can refer to many things (Collins and Ferguson 1993), models consisting of equations are especially common and useful in science and engineering. Thus, Dragoon focuses on such mathematical models.

Dragoon supports modeling of both dynamic and static systems. A system is *dynamic* if it changes over time and *static* if it does not. For instance, a circuit with a battery and a light bulb is a static system because the voltage and current do not change. Conversely, a circuit with a resistor and a charged capacitor in series is a dynamic system because the current starts high and gradually decreases as the capacitor discharges through the resistor.

Mathematical models of dynamic systems are often expressed as sets of differential equations, whereas mathematical models of static system are expressed as sets of algebraic equations. A model of the battery-bulb circuit is *V = I* × *R*, where *V* is the battery voltage, *I* is the current around the circuit, and *R* is the resistance of the bulb. A model of the resistor-capacitor circuit is *dV*/*dt = − 1* × *V*/*RC*, where *V* is the voltage across the capacitor, *C* is the capacitance of the capacitor, and *R* is the resistance of the resistor.

In university courses on electrical engineering, students gradually become skilled at constructing mathematical models of circuits as they work through hundreds of problems. This skill serves them well when they need to understand or design new circuits. However, most other people who need to understand circuits, including the students to be taught by ElectronixTutor, lack such skills in mathematical model construction. Indeed, many people are frustrated even by algebra word problems that can be solved by constructing a mathematical model. In general, it seems doubtful that everyday people, with weak model construction skills, could use mathematical model construction as a method for understanding natural and engineered systems.

The challenge for Dragoon is to make it easy for ordinary people to construct mathematical models that they can use as a way to understand natural and engineered systems. In particular, Dragoon’s role in ElectronixTutor is to help Navy personnel understand analog electronic circuits.

**Fig. 4:**
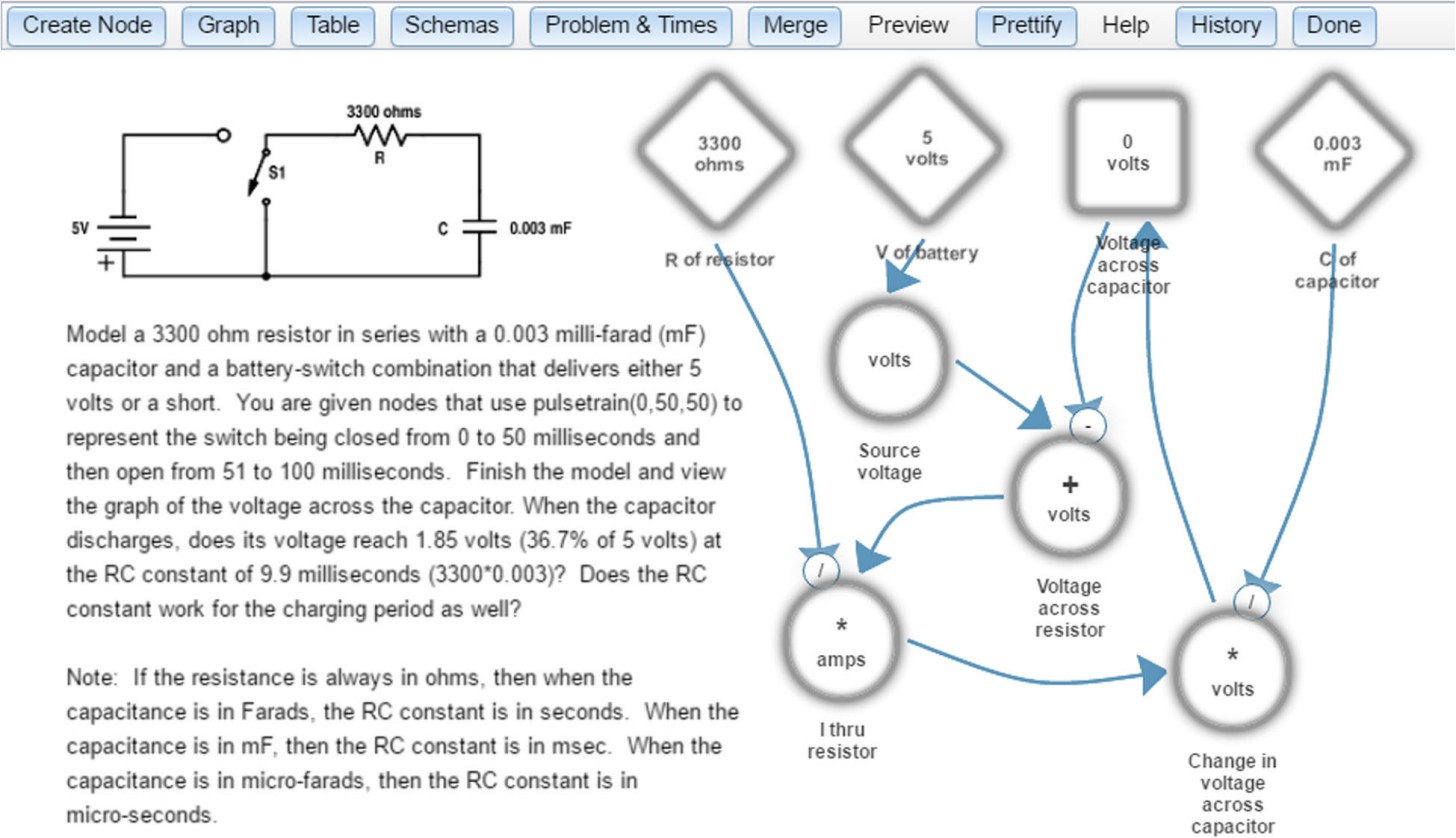
The Dragoon model editor

Figure 4 shows a Dragoon screen. On the left is a problem, which is to construct a model of a simple but realistic resistor-capacitor circuit. On the right are nodes and links that comprise a model of the circuit. Instead of equations, Dragoon has a graphical notation similar to the stock-and-flow notation used by Stella (Doerr 1996), Vensim (VentanaSystems 2015), Powersim (PowerSim 2015), and other dynamic systems modeling environments. In Dragoon, circular nodes represent simple mathematical functions (e.g., the “I thru resistor” is “voltage across resistor” divided by “R of resistor”). Diamond-shaped nodes represent parameters, which are constants whose values can be changed by the user. Square nodes represent accumulators, which integrate (sum up) their inputs over time (e.g., “voltage across capacitor” starts at zero volts and adds in the value of “change in voltage across capacitor” at each millisecond). Dragoon has an editor for creating nodes, entering their mathematical definitions, and describing them in natural language.

**Fig. 5:**
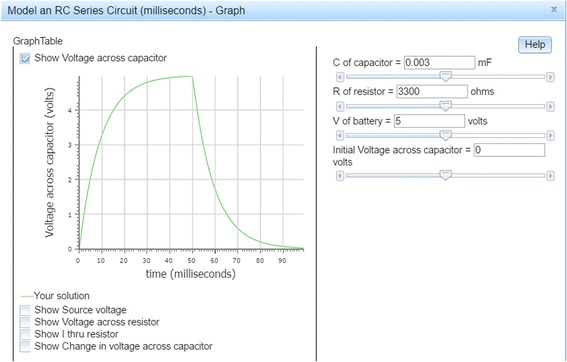
Graphs drawn by Dragoon

When the user clicks on a Graph button, Dragoon displays graphs of the nodes’ values over time (see Fig. 5). Every parameter has a slider for changing its value (shown in the right panel), and the changes are reflected instantly in the graphs of the values of the non-parameters (shown in the left panel).

What has been described so far is just the typical model construction system: an editor for constructing a model and displaying its predictions. When Dragoon is in author mode, those are its main functions. When in student mode, Dragoon can provide four kinds of feedback as students construct a model:

1. *Immediate feedback* mode. Dragoon colors an entry in the node editor green if its value matches the corresponding value in the author’s model and red otherwise. When too many mismatches have been made on an entry, Dragoon provides the correct entry but colors the node yellow. The yellow persists, which discourages guessing.
2. *Coached* mode. Same as immediate feedback mode, except that the students are required to follow a problem-solving strategy that is known to enhance learning (Chi and VanLehn 2010; Wetzel et al. 2016; Zhang et al. 2014).
3. *Delayed feedback* mode. After students have constructed a model, they receive feedback on its predictions, which is presented by drawing the students’ model predictions and the author’s model prediction on the same graph.
4. *No feedback* mode. Students receive no feedback. However, Dragoon still compares their model to the author’s model and updates its assessment of the student’s competence.

Wetzel et al. (2016) provides more detail on how Dragoon instructs students and how it works.

In addition to helping students construct a model, Dragoon has three activities that help students understand a model that has already been constructed by “executing” it in different ways. These activities were developed specifically for use in the ElectronixTutor and have not yet been generalized to other task domains.

**Fig. 6:**
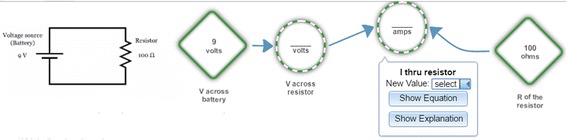
A Dragoon numerical execution activity

Figure 6 shows a numerical execution activity. The student “executes” the model by selecting values for the non-parameter nodes. This helps students practice recalling the formulae inside the nodes.

**Fig. 7:**
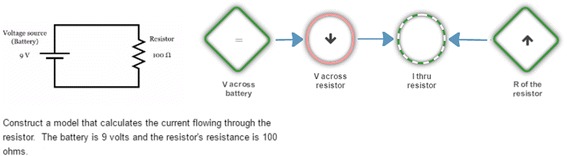
A Dragoon incremental execution activity

Figure 7 shows an incremental execution activity. It helps students obtain a qualitative understanding of the circuit by propagating an upward or downward increment in a parameter’s value through the circuit. This particular problem states that the “V across battery” is constant and “R of the resistor” goes up. The student should say that “V across the resistor” stays the same by choosing “=” from a menu, but the student has instead claimed that the voltage goes down, so Dragoon has given immediate negative (red) feedback.

There is also a waveform activity. From a menu of possible waveforms, the student selects the curve that best approximates the behavior of a node’s value over time.

Dragoon does not have a user manual nor does it require user interface training. Instead, when a feature becomes relevant for the first time, Dragoon pops up a callout that explains the feature. It pops up such explanations several times, with different wording each time, and then stops. Thus, user interface training is embedded in the users’ normal workflow.

Dragoon has been evaluated in several studies, but not yet as part of the ElectronixTutor. In a college class on system dynamics modeling (focused on teaching skill in model construction), one half of the students were randomly assigned to Dragoon and the other half used an ordinary system dynamics editor to complete their homework. Of the students who completed their homework, those who used Dragoon scored significantly higher on the post-test than those in the control condition (VanLehn et al. 2016a, 2016b).

In contrast, a second set of studies focused on using model construction to help students more deeply understand specific naturally occurring systems (VanLehn et al. 2016a, 2016b). All of the studies were experiments, in that they compared high school classes using Dragoon to classes learning the same material without Dragoon. The classes were taught by the regular teachers. However, as small-scale studies that randomly assigned classes instead of students, they could not tightly control all sources of variation. The first study produced null results, but it compared learning across just one class period. The second study in four high school science classes showed that instruction based on an earlier version of Dragoon cost only one extra class period (about 50 min) out of four class periods and was more effective than the same content taught without Dragoon. Dragoon was more effective than the same content taught without Dragoon in a third study in three more high school science classes, where two Dragoon classes and one non-Dragoon class met for the same number of class periods. The effect sizes were moderately large on both an open response test (*d* = 1.00) and a concept mapping task (*d* = 0.49). These high school studies suggest that Dragoon has at least partially succeeded in its challenge, which is to make model construction so easy that it can be used to help students more deeply understand natural and engineered systems.

Dragoon is expected to help students learn analog circuits by offering both practice and embedded (“stealth”) assessment that enables the ElectronixTutor to optimize the students’ practice time. Dragoon’s embedded assessment (student modeling) for electronics is based on fundamental schemas. Each schema pairs a portion of a circuit with a portion of a Dragoon model. For instance, the Ohm’s law schema pairs the resistor with three nodes that implement Ohm’s law (“I thru resistor,” “voltage across resistor,” and “R of resistor”). When an author constructs a problem, the author indicates which nodes go with which schema. When a student is constructing a model or executing a model incrementally, numerically, or as waveforms, Dragoon keeps track of which nodes the student got right on the first attempt. It converts that binary per-node scoring into scores for the schemas and reports these scores to the ElectronixTutor when the problem solving is finished. ElectronixTutor updates its profile of the student and chooses a problem that addresses deficits—schemas that the student has not yet mastered.

Our earlier studies have already shown that Dragoon is sufficiently easy for high school and college students to use so that they can both rapidly acquire skill in model construction and more deeply understand specific natural and engineered systems. When combined with the intelligent task selection of the ElectronixTutor, it is expected to help Navy ATT students learn electronics.

### LearnForm

**Fig. 8:**
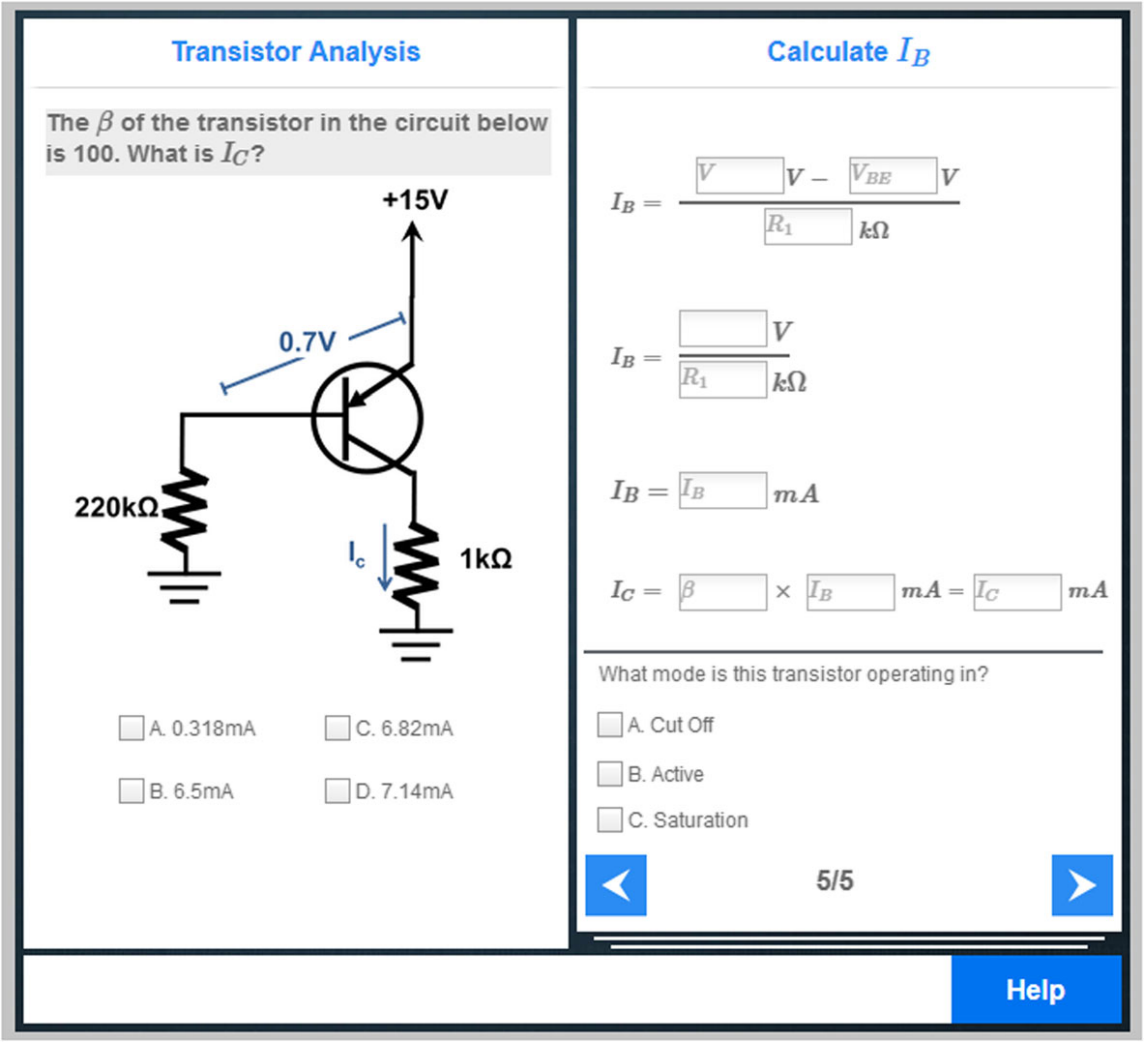
A problem-solving task on LearnForm

LearnForm is a learning platform developed by the Raytheon/BBN team. It is a domain-independent online learning platform that is used for the creation and delivery of problem-solving-based learning tasks. Students learn by solving problems such as the one shown in Fig. 8. A problem starts out with the presentation of the problem statement, shown on the left-hand side of Fig. 8. Although the learning platform supports other forms of responses, all problem statements authored for our Electronics course use a multiple-choice question format.

Students are allowed to solve a given problem without assistance or they can click on the help button, which presents an expert’s decomposition of the problem into a sequence of steps. The problem-solving interface allows free navigation through the steps, i.e., the students are not required to strictly follow the steps. They can choose to skip the current step or revisit previous steps as they find it necessary to help them solve the problem. We consider this as a form of scaffolding achieved through intuitive user interface design. Furthermore, students are not forced to work through every element of every step. Rather, they are allowed free exploration of the problem’s solution to the extent allowed by the pre-designed steps. Feedback is provided for every problem-solving action, and help in the form of hints is available upon request. The learning task concludes when the student inputs the correct answer on the problem statement.

The LearnForm problems available for ElectronixTutor are organized in two different sections: basic circuits and advanced circuits. The basic circuits section includes 46 problems. These were developed by two teachers along with problems in other topics in a high school-level physics course (e.g., electrostatics) that are not part of the target curriculum of ElectronixTutor. The basic circuit problems cover three types of resistor circuits (series, parallel, and complex). An additional 16 problems included in the advanced circuits section were developed by one electronics subject matter expert. These problems introduce transistor- and diode-based circuits, including diode limiters, diode clampers, and five configurations of transistor-based amplifiers (common emitter, common collector, common base, push-pull, and multi-stage).

The teachers as well as the subject matter expert used the *LearnForm* workbench to author these problems. The workbench comprises a WYSIWYG (What You See is What You Get) editor that is used to compose the problem statement and solution steps. From an author’s perspective, statements and steps are fixed-sized tiles. In a manner akin to presentation editing software like PowerPoint and Keynote, these tiles are blank canvases that can be populated with elements like labels, images, text fields, combo-boxes, and option boxes that are available from a palette. The tiles corresponding to solution steps are carefully designed to guide the students through an ideal solution to the problem. The iterative authoring process followed in the development of these problems emphasizes that the ideal sequence of steps is not the one that offers the shortest or fastest solution but one that exercises all of the necessary conceptual knowledge and procedural skills along the way. The workbench also includes a tutor development tool that allows authors to employ a programming-by-demonstration technique without requiring a computer science or cognitive science background. The authors can manually enrich the tutors with text-based feedback and hint prompts.

### ASSISTments

As discussed earlier, ASSISTments has played a system integration role in coordinating learning resources within topic bundles. However, ASSISTments also played an important role in skill building for Ohm’s law and Kirchhoff’s law, which have a mathematical foundation. It is difficult to reason about a circuit if one lacks skill in rudimentary quantitative computations that involve voltage, resistance, current, etc. The ASSISTments research team at WPI took the lead on developing these quantitative skill building modules.

ASSISTments (Heffernan and Heffernan 2014, https://www.assistments.org/) allows teachers to create materials for mathematics (as well as other topics) to see how well students perform and to interact with researchers on possible improvements based on the science of learning. Authoring tools are available to guide the instructors in creating the materials. The *Builder* guides the curriculum designer or teacher in creating lessons, whereas the *Teacher* view shows performance of particular students on particular lessons, and the *Student* view guides the students in completing tasks and viewing feedback on their performance. These three perspectives are extremely important for scaling up a system because it accommodates the points of view of curriculum designers, instructors, and students.

In 2015, ASSISTments was used by over 600 teachers in 43 states and 12 countries, with students completing over 10 million mathematics problems. Learning gains are well-documented and explain the success in the system being scaled up for widespread use. Rochelle et al. (2016) reported that ASSISTments improved mathematics scores reliably with an effect size of *d* = 0.18, which was larger than normal expectations of growth by 75%. The Heffernans were invited by the White House in December, 2016, to present their results (Heffernan and Heffernan 2016).

### BEETLE-II

BEETLE-II is an ITS funded by the Office of Naval Research on basic electricity, electronics, and the fundamentals of circuits (Dzikovska et al. 2014). BEETLE-II had a natural language dialog component, but its strength was its macro-level discourse, which was tied to pedagogical instruction strategies. That is, learning gains were primarily explained by the selection of problems and the discourse/pedagogy that guided the interaction at the macro-level. For example, the major predictors of learning gains consisted of the student predicting the behavior of a circuit, explaining why, observing what happens, and explaining discrepancies between prediction and observation. ElectronixTutor included these curricular components and implemented them with ASSISTments. The BEETLE-II materials were appropriate for beginners who needed to understand series versus parallel circuits, voltage, resistance, and other fundamental principles of simple circuits.

### Point & Query

The Point & Query facility was used in conjunction with the diagrams in the AutoTutor ITS. In this P&Q facility, when the student clicks on a hot spot in the diagram, a menu of questions appears, the student selects a question from the menu, and the answer is presented. For example, when the student clicks on a common-base transistor icon, the questions would include “What is a common-base transistor?” and “What is the difference between a common-base and an emitter?” The student selects a question and a short answer is provided. Computers cannot answer every question a student voluntarily asks so this is an option for curious learners, much like a frequently asked question facility.

The Point & Query component has been shown to increase the frequency and depth of student question asking when suitably engineered. Students ask a remarkably small number of questions and a narrow distribution of questions in most learning environments (Graesser and Person 1994) yet the nature of the questions asked are diagnostic of student understanding (Graesser and Olde 2003; Person et al. 1994). Point & Query increases the frequency of student questions in hypermedia environments by one to two orders of magnitude compared to a classroom. It increases the depth of questions (e.g., why, how, and what-if rather than who, what, when, and where) when given suitable learning objectives to create mental models of the subject matter (Graesser et al. 1993).

### Readings and videos

ElectronixTutor has a simplified summary version of each of the topics for trainees to read at their own pace. More in-depth technical material can be accessed and read from the Navy Electricity and Electronics Training Series (U.S. Navy 1998). PowerPoint presentations from ATT training and videos on how to use Dragoon are also available. These learning resources are either suggested as options by the Recommender System or are available when a self-regulated learner wishes to view them.

According to Chi’s interactive-constructive-active-passive (ICAP) framework (Chi 2009; Chi and Wylie 2014), there are four modes of cognitive engagement based on students’ overt behaviors. Learners engage *passively* when they receive information without demonstrating obvious behavior related to learning. They engage *actively* when there is behavior that does not go beyond the information presented (e.g., writing verbatim notes, underlining). They engage *constructively* when behaviors involve generating ideas that go beyond the to-be-learned information, such as reasoning and generating explanations. They engage *interactively* through dialog with a person or digital system that involves constructive activities, such as asking and answering questions with a peer and defending a position in an argument. Sometimes, it is necessary for the student to read documents or view videos, which are normally associated with passive learning rather than active, constructive, and interactive learning resources. However, these learning resources can be accompanied by more active forms of learning, as in the case of note taking and drawing diagrams (Chi and Wylie 2014).

### Data analyses through LearnSphere

The data collected from ElectronixTutor is sizeable because it includes diverse learning resources, knowledge components, and learners. The student model data and history of the tutorial experiences are stored in the Learning Record Repository. These data need to be analyzed with statistical, mathematical, and computational modeling that is performed by researchers at many institutions. To coordinate these data analyses, development and efficacy data for the ElectronixTutor project will be stored, shared, and analyzed with *LearnSphere* (Stamper et al. 2016).

LearnSphere builds on the Pittsburgh Science of Learning Center’s *DataShop* (Koedinger et al. 2010), the world’s largest open repository of learning transaction data, and *MOOCdb* (Veeramachaneni et al. 2013), a database design and supporting framework created to harness the vast amounts of data being generated by Massively Open On-line Courses (MOOCs). LearnSphere integrates existing and new educational data infrastructures to offer a world-class repository of education data. LearnSphere will enable new opportunities for learning scientists, course developers, and instructors to better evaluate claims and perform data mining. A standard set of analysis tools allows researchers to readily perform quantitative analyses and to observe workflows of fellow researchers. By using a community-based tool repository, researchers will be able to quickly build new models, create derivative works, improve existing tools, and share their work with their team and other teams.

## Conclusions

### Next steps in assessing ElectronixTutor

Now that we have successfully integrated all of the distinct ITSs into a fully functional ElectronixTutor prototype, the teams are in the process of testing and revising the system. Pilot testing has begun with engineering students to provide user feedback. Numerous lay and professional electrical engineering educators have examined the system and provided extensive feedback. Plans are underway for testing ElectronixTutor on engineering students at the University of Memphis and Florida Institute of Technology to assess its impact on learning gains, based on a test that assesses the learning of approximately 80 knowledge components. ElectronixTutor will be a supplement to the normal university courses and possibly become part of the curriculum. We plan on having ElectronixTutor available to sailors for training in their classes by fall of 2018.

At this point, we have made principled decisions on what learning resources to recommend to students at the right time. These begin with a topic of the day, guided by the curriculum calendar. Also available are a small number of recommended topics for consideration by the student, based on the global history of the student’s performance on knowledge components as well as other cognitive and non-cognitive characteristics. For each topic, the learning resources are locally organized by topic bundles that assign learning resources adaptively based on the student’s immediate performance on that topic. ElectronixTutor also allows access to all of the topics and learning resources for consideration for those students who are self-regulated learners.

The above organization on ElectronixTutor will no doubt need to be revised as data are collected on performance and aptitude-treatment interactions. We expect the more knowledgeable students to benefit from the broad deep-reasoning questions of AutoTutor and the mental model constructions of Dragoon. Clearly, it would be beyond the beginning student’s zone of proximal development to attempt these difficult problems. At the other end of the performance distribution, the low-knowledge students will presumably benefit from reading, the skill building tests of ASSISTments, and the basic lessons on simple circuits provided by BEETLE-II. The knowledge check questions of AutoTutor and the LearnForm problems are suited to trainees with an intermediate level of knowledge. These are principled assignments of learning resources that are based on student performance, but it remains to be seen whether these principles are confirmed by empirical data.

At this point, we are uncertain whether a given trainee might benefit from one type of learning resource over others and also whether some knowledge components are best acquired by a particular learning resource. For example, perhaps one trainee benefits most from the verbal reasoning of AutoTutor but another trainee benefits most from the visualizations of Dragoon. Although evidence of learning styles is empirically questionable (Pashler et al. 2008; Rohrer and Pashler 2012), perhaps such tailored learning resources may pan out. Alternatively, a mixture of learning principles may make sense, following the principles of cognitive flexibility (Spiro et al. 1992) and encoding variability (Bjork and Allen 1970). We plan to explore and discover such relationships through data mining methodologies (Baker 2015), followed by controlled experiments to test promising trends.

The role of motivation and emotions is also expected to play an important role in the long-term evolution of ElectronixTutor (see the micro-level metrics in the Appendix). These motivational and affective states can to some extent be identified by the patterns and timing of conversation and human-computer interaction in addition to facial expressions, body posture, and other sensory channels that are available to the ITS (D’Mello et al. 2009; D’Mello and Graesser 2012). Algorithms are available for tracking the extent to which the student has perseverance or grit, which we consider a predominantly positive attribute, but also incurs a potential cost of wheel spinning (Beck and Gong 2013). Confusion is known to have positive benefits when there is productive thought (D’Mello et al. 2014), but protracted confusion is undesirable. Boredom and disengagement are of course incompatible with learning and should prompt the ITS to change gears and present a different topic or a different difficulty level. Frustration is generally undesirable but might fuel sustained concentration by the most accomplished students. When these various affective states are recognized by ElectronixTutor, there needs to be principled ways for the tutor to respond. Previous studies with AutoTutor have indeed confirmed that learning can improve when the system responds to the affective states of the learner (D’Mello and Graesser 2012). However, the field is in its infancy in discovering and testing such interactions between affective states, mastery levels, and tutoring strategies.

In closing, we consider it a milestone to integrate multiple ITS learning resources in a single ElectronixTutor system. It permits an eclectic strategic approach to training students with idiosyncratic histories and psychological characteristics. If one ITS module does not work well, there are many others to try out. This is a substantively different approach than forcing a single ITS mechanism on everyone.

## Appendix

**Table 1:** Inventory of ITS messages aggregated from ITS design teams

Learner performance messages		
Completed	User completed a learning task was completed and a certain score (0–1) was returned.	
KnowledgeComponentScore	User demonstrated mastery of a specific knowledge component on a task (0–1)	
CompletedAllSteps	User completed a certain percentage of all steps of a task (0–1)	
CompletedStep	User completed a specific step of a task (0–1 for quality of step)	
AnswerSemanticMatch	User’s answer matched the ideal to a certain quality level (0–1)	
Misconception	User demonstrated a known misconception	
Help messages		
TaskSupport		User received a certain overall level of help on a task (0–1)
TaskHelp		User received some kind of help on a certain task step
TaskHint		User received a hint on a certain task step
TaskFeedback		User received feedback (positive, negative, or neutral) on a certain step
TaskDecomposition		User decomposed a task into subparts to complete (used only by the LearnForm ITS)
TaskHelpCount		Number of times user requested help in a session (summary, for systems that cannot report individual events)
User interface communication messages		
Presented		Information was presented to the user by the system
SelectedOption		User selected a given option in the system
SubmittedAnswer		User submitted a certain answer (e.g., text, option choice)
System control messages		
Loaded		The task loaded successfully and is running
Heartbeat		The task is still running and has not frozen or otherwise stopped
Micro-level metrics messages		
WordsPerSecond		System estimated user input text as a given words per second
ActionsPerSecond		System estimated user input actions per second
Persistence		System estimated persistence on a given task
	Impetuousness	System estimated impetuousness (e.g., guessing) on a task
	GamingTheSystem	System estimated gaming the system on a task
	WheelSpinning	System estimated wheel spinning on a given task
	Confusion	System estimated user confusion on a given task
	Disengagement	System estimated user disengagement on a given task
